# Work engagement and its association with obesity and lifestyle factors in a large working population: a sex-stratified analysis

**DOI:** 10.3934/publichealth.2026017

**Published:** 2026-03-05

**Authors:** Lucía Garrido Sepúlveda, Pedro Juan Tárraga López, María Teófila Vicente-Herrero, Lluis Rodas Cañellas, Ángel Arturo López González, José Ignacio Ramírez-Manent

**Affiliations:** 1 Home Hospitalization Unit, Arnau de Vilanova Hospital, Valencia, Spain; 2 Faculty of Medicine, University of Castilla-La Mancha, Albacete, Spain; 3 ADEMA University School, Balearic Islands, Spain; 4 Palma de Mallorca Health Service, Balearic Islands, Spain; 5 Faculty of Medicine, University of the Balearic Islands, Palma, Spain

**Keywords:** work engagement, obesity, visceral fat, life style, occupational health, psychosocial factors

## Abstract

**Background:**

Work engagement, defined as a positive, fulfilling psychological state characterized by vigor, dedication, and absorption, has been proposed as a potential protective factor for cardiometabolic health. However, its relationship with obesity—particularly visceral adiposity—remains poorly understood. This study aimed to examine the association between work engagement and different adiposity indicators and to explore the mediating role of lifestyle behaviors in a large sample of Spanish workers.

**Methods:**

A cross-sectional analysis was conducted among employees attending occupational health evaluations between 2021 and 2022. After applying inclusion and exclusion criteria, 111,612 participants (60.1% men) were included. Work engagement was assessed using the Utrecht Work Engagement Scale (UWES-9) and categorized into very high, high, moderate, and low levels. Obesity and adiposity were evaluated through BMI, waist-to-height ratio (WtHR), CUN-BAE, and METS-VF. Logistic regression models estimated odds ratios (OR) and 95% confidence intervals (CI) for obesity outcomes across engagement levels, with progressive adjustment for sociodemographic and lifestyle covariates. Mediation analyses evaluated the indirect effects of physical activity, Mediterranean diet adherence, and smoking.

**Results:**

Lower engagement levels were independently associated with higher odds of adiposity across all indices, particularly visceral fat (METS-VF). Participants with low engagement had an adjusted OR = 2.59 (95% CI 2.34–2.85) for high METS-VF compared with those with very high engagement (p-trend < 0.001). Approximately 40% of this association was mediated by lifestyle factors, mainly physical activity. The results remained robust across sensitivity analyses, including nonlinear modeling, imputation, and cluster adjustment.

**Conclusions:**

Lower work engagement is strongly associated with increased visceral adiposity among Spanish workers, partly through behavioral mechanisms. Enhancing engagement may represent an effective psychosocial strategy to improve both psychological well-being and metabolic health in occupational settings.

## 1. Introduction

Obesity remains one of the leading global health challenges, with prevalence increasing in nearly all regions and age groups, with substantial consequences for cardiovascular, metabolic, and psychosocial health [1]. Beyond biological and environmental determinants, there is growing recognition of psychosocial and behavioral drivers that modulate daily health habits and influence obesity risk [2]. Among these, work engagement—a positive, fulfilling, work-related state of mind characterized by vigor, dedication, and absorption—has gained attention as a potential determinant of well-being and health outcomes [3]. The Job Demands–Resources (JD-R) model posits that engagement arises when job and personal resources exceed demands, fostering adaptive coping, positive affect, and resilience, which may indirectly reduce physiological stress and unhealthy behaviors [4].

Empirical research has largely focused on engagement in relation to mental health, productivity, and burnout prevention [5],[6]. High levels of work engagement are consistently associated with lower burnout, greater job satisfaction, improved performance, and reduced absenteeism [7],[8]. However, emerging evidence suggests that engagement may also play a role in physical health and metabolic regulation, acting through behavioral pathways such as increased physical activity, better dietary habits, and reduced smoking [9]. In a meta-analysis, higher engagement was linked to more frequent leisure-time physical activity and lower sedentary time [10], while recent reviews highlight the bidirectional relationship between work engagement and health-promoting behaviors in diverse occupational groups [11].

A recent cross-sectional study conducted in an Italian population of administrative employees highlights the key role of leisure-time physical activity in reducing musculoskeletal disorders, enhancing work engagement, and preventing burnout. The analysis of emotional exhaustion is consistent with previous research on burnout and suggests a potential time-dependent cascade effect. This study further indicates that physical activity promotes work engagement beyond the levels currently recommended [12].

Lifestyle behaviors are critical intermediaries between psychosocial well-being and physical health [13]. The IPAQ-SF has been validated in the Spanish adult and working population, showing adequate reliability and construct validity [14],[15]. The 14-item Mediterranean Diet Adherence Screener (MEDAS), originally developed for the PREDIMED trial, offers a simple, validated measure of adherence to a cardioprotective dietary pattern [16]. These instruments, widely used in occupational cohorts, enable reliable evaluation of modifiable health behaviors that may mediate the relationship between engagement and obesity.

Traditional measures of adiposity, such as the Body Mass Index (BMI), though simple and widely used, fail to capture body fat distribution and visceral adiposity, which are more metabolically active and strongly associated with cardiometabolic risk [17]. Alternative indices, including waist-to-height ratio (WtHR) and the Clínica Universidad de Navarra Body Adiposity Estimator (CUN-BAE), have demonstrated better predictive accuracy for metabolic syndrome and diabetes [18],[19]. More recently, the Metabolic Score for Visceral Fat (METS-VF) has emerged as a surrogate marker for visceral adiposity, integrating anthropometric and biochemical variables. METS-VF correlates strongly with insulin resistance, metabolic syndrome, and cardiovascular outcomes [20]–[22]. Its use in occupational epidemiology provides a refined estimation of metabolic risk beyond conventional anthropometric markers.

From a theoretical perspective, work engagement may play a protective role against obesity by being associated with lower levels of chronic stress, healthier lifestyle behaviors, and enhanced self-regulatory capacity, thereby acting as a potential mediator between psychosocial working conditions and metabolic health outcomes.

Despite robust theoretical links, the empirical relationship between work engagement and obesity remains poorly characterized. Only a few studies have examined how psychological engagement relates to body composition, and none have systematically addressed the behavioral mediation of this association. Scientific evidence indicates that higher psychological commitment is associated with more favorable body composition indicators by promoting adherence to regular physical activity. Elevated levels of motivation and behavioral engagement are linked to lower body fat, improved body mass index, and greater fat-free mass, with indirect positive effects observed following psychological interventions [23].

Given that adults spend a substantial portion of their lives in work environments where psychosocial factors influence lifestyle choices, understanding how engagement interacts with adiposity could inform preventive strategies. Available studies suggest a consistent, albeit indirect, relationship between work engagement and body composition or closely related variables. Evidence indicates that more favorable body composition, higher levels of physical activity, and a positive body image are associated with lower stress levels, reduced absenteeism, and greater psychological well-being, all of which contribute to enhanced work engagement. Although most studies are observational, they highlight physical health as a relevant determinant of work engagement [24]–[27].

Therefore, the present study aims to examine the association between work engagement (UWES-9) and multiple adiposity indices—BMI, WtHR, CUN-BAE, and METS-VF—in a large cohort of Spanish workers. We hypothesize that (i) lower engagement will be associated with higher odds of obesity across all indices, with the strongest associations for visceral fat (METS-VF); and (ii) this relationship will be partially mediated by lifestyle behaviors, particularly physical activity, Mediterranean diet adherence, and smoking. By integrating psychosocial theory, validated behavioral instruments, and advanced adiposity metrics, this research contributes novel evidence on the intersection between occupational well-being and metabolic health.

## 2. Methods

### 2.1. Study design and population

This cross-sectional study was conducted within a large cohort of Spanish employees who underwent routine occupational health examinations between January 2021 and December 2022. Prior to enrolment, all participants provided written informed consent acknowledging their voluntary involvement in the study. All assessments were performed by trained healthcare personnel following standardized clinical protocols in accredited occupational health centers across Spain. Data collection included anthropometric measurements, biochemical profiles, sociodemographic characteristics, and self-reported lifestyle and psychosocial variables. Data were obtained from a network of accredited Occupational Health Services operating across multiple regions of Spain. The study population comes from workers from the Spanish autonomous communities (Balearic Islands, Andalusia, Canary Islands, Valencian Community, Catalonia, Madrid, Castilla-La Mancha, Castilla y León, Basque Country). These services provide mandatory periodic health examinations to employees from a wide range of economic sectors, including industry, construction, transportation, healthcare, education, retail, and administrative services. This nationwide coverage ensures that the dataset reflects the heterogeneity of the Spanish working population and enhances the external validity and reproducibility of the study. All employees attending mandatory periodic occupational health examinations during the study period were eligible for inclusion.

Exclusion criteria were defined a priori and included (a) missing data in key sociodemographic, anthropometric, or lifestyle variables; (b) implausible anthropometric or biochemical values, operationalized as >4 standard deviations from the sample mean; and (c) self-reported diagnosis of major chronic diseases (type 2 diabetes, cardiovascular disease, cancer, or severe metabolic disorders) to avoid reverse causation.

Data were collected through standardized protocols used across accredited Occupational Health Services nationwide. These protocols included uniform procedures for anthropometry, blood sampling, interview-based lifestyle assessment, and administration of validated questionnaires. All measurements were performed by trained healthcare professionals following harmonized quality-control criteria.

The participant selection process followed a prespecified flow: all eligible workers were screened (N = 113,051), exclusion criteria were applied, and the final analytic sample consisted of 111,612 workers (60.1% men, 39.9% women), aged 18–69 years.

Given the observational nature of the study and the availability of a large existing cohort, no a priori sample size calculation was performed. However, a post hoc power assessment indicated that with a final sample of 111,612 participants and an outcome prevalence of approximately 30%, the study had >99% power to detect small effect sizes (minimum detectable odds ratio ≈ 1.10) across the four work engagement categories, assuming a two-sided α = 0.05. Therefore, the sample size was more than sufficient for the primary analyses.

### 2.2. Anthropometric and metabolic measurements

Body weight, height, and waist circumference were obtained following standard procedures, with participants wearing light clothing and no shoes. From these measurements, four adiposity indices were derived:

1. Body Mass Index (BMI) = weight (kg)/height² (m²); obesity defined as BMI ≥ 30 kg/m².

2. Waist-to-height ratio (WtHR) = waist (cm)/height (cm); threshold ≥ 0.5 indicating elevated cardiometabolic risk.

3. Clínica Universidad de Navarra Body Adiposity Estimator (CUN-BAE), estimating body fat percentage using sex-, age-, and BMI-adjusted equations; obesity defined as ≥25% for men and ≥35% for women [28].

4. Metabolic Score for Visceral Fat (METS-VF), a validated proxy of visceral adiposity combining anthropometric and metabolic parameters (BMI, triglycerides, glucose, HDL-c, and WtHR). Cutoff points were ≥7.18 for men and ≥6.86 for women [29].

### 2.3. Sociodemographic and lifestyle variables

Sociodemographic data included sex, age, and occupational social class, classified according to the Spanish National Classification of Occupations (CNO-11) and grouped into high (class I), medium (class II), and low (class III) categories [30].

Lifestyle-related variables were assessed using standardized, validated instruments routinely administered during occupational health examinations. All questionnaires were completed under the supervision of trained medical staff to minimize recall bias and ensure consistency across centers.

• Smoking status was categorized as current smoker (regular tobacco consumption at the time of examination) or non-smoker (including former smokers and never smokers). Smoking was self-reported through a structured question validated in the Spanish National Health Survey, with periodic cross-verification by cotinine testing in a subsample for quality assurance.

• Physical activity was evaluated using the International Physical Activity Questionnaire—Short Form (IPAQ-SF) [31], which quantifies the frequency and duration of walking, moderate, and vigorous activities over the preceding seven days. Total activity was expressed in metabolic equivalent minutes per week (MET-min/week), and participants were classified according to World Health Organization (WHO) recommendations: sufficiently active (≥600 MET-min/week) or insufficiently active (<600 MET-min/week). The IPAQ-SF has demonstrated good reliability and validity for population-based surveillance, with an intraclass correlation coefficient of 0.76 in Spanish adults.

• Dietary habits were assessed with the 14-item Mediterranean Diet Adherence Screener (MEDAS) developed by the Prevención con Dieta Mediterránea (PREDIMED) study. The questionnaire evaluates adherence to key dietary principles such as olive oil consumption, fruit and vegetable intake, red meat limitation, and moderate wine consumption. Each item scores one point for adherence, yielding a total range of 0–14. A score of ≥9 was considered indicative of adequate adherence to the Mediterranean diet, consistent with prior validation studies in Mediterranean populations [32].

### 2.4. Assessment of work engagement

Work engagement was evaluated using the Utrecht Work Engagement Scale (UWES-9) [33], a widely validated instrument that captures three core dimensions of engagement: vigor, dedication, and absorption. Total scores were computed and categorized into four levels (very high, high, moderate, and low) based on validated percentile cutoffs established for Spanish working populations [34]. The UWES-9 demonstrated excellent internal consistency (Cronbach's α = 0.92) and construct validity in this cohort.

From a psychometric standpoint, the Utrecht Work Engagement Scale (UWES-9) is the most widely used instrument to measure engagement. It captures the three core dimensions—vigor, dedication, and absorption—and has shown excellent internal consistency (Cronbach's α ≈ 0.90) and factorial validity across languages and occupational settings [35],[36]. Recent validations in Thailand, China, and Europe confirmed the robustness of the UWES-9 structure in healthcare and service sectors, consolidating its utility for large-scale epidemiologic studies [36],[37]. Work engagement was assessed using the Spanish validated version of the Utrecht Work Engagement Scale (UWES-9), which has shown strong psychometric properties, including high internal consistency and construct validity among Spanish workers [33],[38].

### 2.5. Statistical analysis

Descriptive statistics were expressed as means (standard deviations) or frequencies (percentages), and between-group differences were evaluated using Student's t-test or χ² test as appropriate. Logistic regression models were used to examine the association between work engagement (very high, high, moderate, and low) and obesity according to four adiposity indices: BMI, WtHR, CUN-BAE, and the METS-VF. A stepwise adjustment strategy was applied: Model 1 included age and sex; Model 2 additionally included social class; Model 3 incorporated physical activity, Mediterranean diet adherence, and smoking; and Model 4 represented the fully adjusted model. Odds ratios (OR) and 95% confidence intervals (CI) were reported, with p for trend estimated by treating engagement as an ordinal variable.

Nonlinearity for continuous covariates (age and, where relevant, blood pressure and lipid levels) was assessed using restricted cubic splines with three to five knots. Variance inflation factors (VIF) were calculated to test for multicollinearity, and values above 10 were considered indicative of concern. Model calibration and discrimination were evaluated by Brier score, calibration slope and intercept, and area under the receiver operating characteristic curve (AUC). Clustered data were accounted for using robust standard errors and mixed-effects logistic models with random intercepts by company to control for intragroup correlation. Missing data were addressed via multiple imputation by chained equations (≥20 imputations), and results were compared with complete-case analyses.

Sensitivity analyses included repeating models with each adiposity index as a continuous z-score using linear or quantile regression and re-running the main models after excluding current smokers. Subgroup analyses were performed by sex, age group, social class, physical activity, and Mediterranean diet adherence, and effect modification was tested using multiplicative interaction terms. To account for multiple testing, the false discovery rate was controlled using the Benjamini–Hochberg procedure in secondary analyses. Causal mediation analyses were conducted within a counterfactual framework to estimate natural direct and indirect effects of engagement on visceral adiposity (METS-VF) through three parallel behavioral mediators: physical activity, Mediterranean diet adherence, and smoking. Proportions mediated were derived using a nonparametric bootstrap with 5000 replications. E-values were computed to evaluate potential unmeasured confounding.

Given the large sample size, we calculated effect sizes alongside p-values to better assess the practical relevance of group differences. Standardized mean differences (Cohen's d) were computed for continuous variables, and Cramer's V for categorical variables. Effect sizes were interpreted according to Cohen's conventional benchmarks: for standardized mean differences (Cohen's d), values of 0.20, 0.50, and 0.80 were considered small, moderate, and large effects, respectively; for Cramer's V, values of approximately 0.10, 0.30, and 0.50 indicated small, moderate, and large associations. These thresholds were used as interpretative guidelines rather than strict cutoffs [39].

Given its higher metabolic specificity and lower collinearity, advanced analyses (nonlinear modeling, mediation, calibration, and sensitivity diagnostics) were performed primarily using METS-VF. Statistical significance was defined as p < 0.05 (two-tailed). Analyses were performed using SPSS version 29.0 (IBM Corp., Armonk, NY) and R version 4.3.1 (R Foundation for Statistical Computing, Vienna, Austria).

## 3. Results

The study population comprised workers from a wide variety of occupational sectors (e.g., industry, construction, transport, healthcare, education, retail, and administrative roles), making the sample broadly representative of the Spanish working population.

**Table 1.** Baseline characteristics of the study population.

Variable	Men n = 67,126	Women n = 44,486	p-value	Effect size

	Mean (SD)	Mean (SD)		
Age (years)	39.8 (10.3)	39.2 (10.2)	<0.001	0.07^a^
Height (cm)	174.0 (7.1)	161.2 (6.6)	<0.001	1.88^a^
Weight (kg)	81.1 (13.9)	65.3 (13.2)	<0.001	1.17^a^
Waist circumference (cm)	87.7 (9.2)	73.9 (7.9)	<0.001	1.60^a^
Hip circumference (cm)	100.1 (8.4)	97.2 (9.0)	<0.001	0.33^a^
Systolic blood pressure (mmHg)	124.4 (15.1)	114.3 (14.7)	<0.001	0.67^a^
Diastolic blood pressure (mmHg)	75.4 (10.6)	69.6 (10.3)	<0.001	0.55^a^
Cholesterol (mg/dL)	195.8 (38.8)	194.0 (36.6)	<0.001	0.05^a^
HDL-cholesterol (mg/dL)	51.0 (7.0)	53.7 (7.6)	<0.001	0.37^a^
LDL-cholesterol (mg/dL)	120.3 (37.7)	122.6 (37.2)	<0.001	0.06^a^
Triglycerides (mg/dL)	123.7 (88.6)	88.1 (46.3)	<0.001	0.49^a^
Glucose (mg/dL)	88.1 (13.0)	84.1 (11.6)	<0.001	0.32^a^
	n (%)	n (%)		
Age group <30 years	12,046 (17.9%)	8639 (19.4%)	<0.001	0.04^b^
30–39 years	22,091 (32.9%)	14,890 (33.5%)	0.07	0.01^b^
40–49 years	19,944 (29.7%)	12,999 (29.2%)	0.15	0.01^b^
50–59 years	11,059 (16.5%)	6813 (15.3%)	<0.001	0.02^b^
60–69 years	1986 (3.0%)	1145 (2.6%)	<0.001	0.01^b^
Social class I	3586 (5.3%)	3098 (7.0%)	<0.001	0.07^b^
Social class II	11,817 (17.6%)	14,870 (33.4%)	<0.001	0.18^b^
Social class III	51,723 (77.1%)	26,518 (59.6%)	<0.001	0.19^b^
Physical activity (yes)	30,722 (45.8%)	23,080 (51.9%)	<0.001	0.06^b^
Mediterranean diet (yes)	27,629 (41.2%)	22,761 (51.2%)	<0.001	0.10^b^
Smokers	24,881 (37.1%)	14,535 (32.7%)	<0.001	0.05^b^
Engagement: low	5481 (8.2%)	2436 (5.5%)	<0.001	0.07^b^
Engagement: moderate	29,410 (43.8%)	19,377 (43.6%)	0.42	0.01^b^
Engagement: high	18,044 (26.9%)	8289 (18.9%)	<0.001	0.10^b^
Engagement: very high	14,191 (21.1%)	14,384 (32.3%)	<0.001	0.13^b^

Note: Values are presented as mean (SD) for continuous variables and n (%) for categorical variables. ^a^ Effect size expressed as Cohen's d. ^b^ Effect size expressed as Cramer's V. Effect sizes were interpreted according to Cohen's criteria (small, moderate, large).

**Table 2.** Distribution of engagement categories and obesity indices.

	Number of people	BMI obesity	WtHR high	CUN-BAE obesity	METS-VF high

	n	%	%	%	%
Men
<30 years	12,046	10.5	31.2	22.8	3.5
30–39 years	22,091	16.8	43.5	43.7	7.3
40–49 years	19,944	22.7	54.0	63.7	11.4
50–59 years	11,059	27.1	60.0	78.3	20.4
60–69 years	1986	28.6	66.9	88.0	29.7
Social class I	3586	17.6	43.9	51.8	6.9
Social class II	11,817	17.7	44.6	51.9	9.1
Social class III	51,723	20.0	48.8	53.2	11.8
Yes physical activity	30,722	7.2	14.9	19.9	3.1
No physical activity	36,404	32.8	82.8	80.7	15.9
Yes Mediterranean diet	27,629	8.9	19.8	19.6	3.9
No Mediterranean diet	39,497	30.1	76.3	76.2	13.9
Smokers	24,881	21.0	49.9	56.7	10.2
Non-smokers	42,245	16.9	44.4	46.4	7.7
Engagement low	5481	39.9	69.8	77.2	17.8
Engagement moderate	29,410	22.9	57.8	60.6	13.5
Engagement high	18,044	20.4	30.2	48.8	9.2
Engagement very high	14,191	10.5	18.8	33.9	7.1
Women
<30 years	8639	10.0	11.0	24.0	0.3
30–39 years	14,890	12.8	13.7	35.8	0.6
40–49 years	12,999	16.9	18.2	55.4	1.2
50–59 years	6813	21.6	22.7	77.2	1.9
60–69 years	1145	25.9	27.5	90.5	2.8
Social class I	3098	9.3	10.6	32.8	1.1
Social class II	14,870	10.0	11.5	36.6	1.3
Social class III	26,518	18.7	19.5	54.5	1.9
Yes physical activity	23,080	6.3	7.5	17.8	0.5
No physical activity	21,406	24.7	25.8	78.4	2.9
Yes Mediterranean diet	22,761	7.9	8.8	21.1	0.7
No Mediterranean diet	21,725	22.2	23.3	74.1	2.4
Smokers	14,535	16.6	16.9	50.2	2.1
Non-smokers	29,951	12.1	14.8	40.4	1.8
Engagement: low	2436	30.9	34.9	70.4	3.9
Engagement: moderate	19,377	24.6	27.2	58.6	3.0
Engagement: high	8289	13.0	16.8	42.3	1.9
Engagement: very high	14,384	9.9	13.5	30.4	1.0

Note: BMI, Body Mass Index. WtHR, waist-to-height ratio. CUN-BAE, Clínica Universitária de Navarra Body Adiposity Estimator. METS-VF, Metabolic Score for Visceral Fat.

### 3.1. Descriptive results

Tables 1 and 2 present the descriptive characteristics of the study population. Workers with lower engagement levels exhibited higher adiposity indicators, less physical activity, and lower adherence to the Mediterranean diet. The prevalence of obesity, whether defined by BMI, WtHR, CUN-BAE, or METS-VF, increased progressively across decreasing engagement categories. These trends were consistent in both men and women, though absolute prevalence was higher among males. Although statistically significant due to the large sample size, the difference in age between groups corresponded to a very small effect size (Cohen's d = 0.07), suggesting negligible practical relevance.

### 3.2. Multivariable logistic regression models (sequential adjustment strategy)

The associations between work engagement and each adiposity indicator were examined using a series of sequentially adjusted logistic regression models, following the analytical strategy described in the Methods section (see Table 3). Model 1 was adjusted for age and sex; Model 2 additionally included occupational social class; Model 3 further incorporated lifestyle factors (physical activity, Mediterranean diet adherence, and smoking); and Model 4 represented the fully adjusted model. This stepwise approach was applied consistently to all four adiposity indicators (BMI, WtHR, CUN-BAE, and METS-VF).

**Figure 1.**
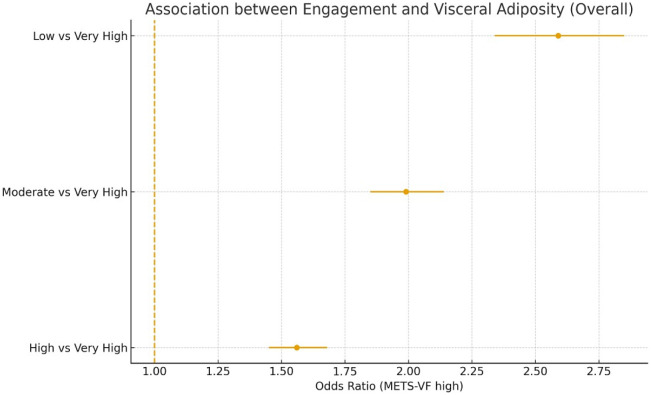
Association between engagement and visceral adiposity (overall METS-VF).

In the fully adjusted model (Model 4), compared with very high engagement, high, moderate, and low engagement levels were associated with progressively higher odds of visceral adiposity (METS-VF > threshold): ORs 1.56 (95% CI 1.45–1.68), 1.99 (1.85–2.14), and 2.59 (2.34–2.85), respectively (p-trend < 0.001). Comparable but slightly lower magnitudes were found for BMI, WtHR, and CUN-BAE. These patterns remained stable after mutual adjustment for behavioral factors (Figure 1).

**Table 3.** Sequentially adjusted multivariable logistic regression models (Models 1–4) for the association between work engagement levels and adiposity indices.

Engagement level (ref: very high)	Model 1 OR (95% CI)	Model 2 OR (95% CI)	Model 3 OR (95% CI)	Model 4 OR (95% CI)
Outcome: BMI obesity
High	1.62 (1.55–1.69)	1.56 (1.49–1.64)	1.50 (1.43–1.58)	1.49 (1.42–1.57)
Moderate	2.32 (2.17–2.48)	2.22 (2.07–2.38)	2.14 (1.99–2.30)	2.11 (1.96–2.27)
Low	2.88 (2.65–3.14)	2.71 (2.48–2.96)	2.58 (2.36–2.82)	2.52 (2.30–2.75)
Outcome: waist-to-height ratio (WtHR ≥ 0.5)		
High	1.48 (1.40–1.56)	1.44 (1.36–1.52)	1.40 (1.32–1.48)	1.38 (1.30–1.47)
Moderate	2.05 (1.93–2.18)	1.98 (1.86–2.11)	1.92 (1.80–2.05)	1.89 (1.77–2.01)
Low	2.63 (2.38–2.91)	2.49 (2.26–2.75)	2.41 (2.18–2.67)	2.35 (2.03–2.67)
Outcome: CUN-BAE obesity
High	1.53 (1.47–1.59)	1.50 (1.44–1.56)	1.46 (1.41–1.52)	1.44 (1.39–1.49)
Moderate	2.08 (1.96–2.21)	2.01 (1.89–2.14)	1.97 (1.85–2.09)	1.92 (1.80–2.05)
Low	2.60 (2.43–2.78)	2.46 (2.29–2.64)	2.40 (2.24–2.57)	2.33 (2.18–2.49)
Outcome: high visceral adiposity (METS-VF)		
High	1.71 (1.60–1.83)	1.65 (1.54–1.77)	1.59 (1.48–1.71)	1.56 (1.45–1.68)
Moderate	2.29 (2.14–2.45)	2.17 (2.03–2.33)	2.05 (1.91–2.21)	1.99 (1.85–2.14)
Low	3.01 (2.74–3.30)	2.84 (2.59–3.11)	2.70 (2.45–2.97)	2.59 (2.34–2.85)

Note: BMI, Body Mass Index. WtHR, waist-to-height ratio. CUN-BAE, Clínica Universitária de Navarra Body Adiposity Estimator. METS-VF, Metabolic Score for Visceral Fat. OR, odds ratio. CI, confidence interval. Model 1: adjusted for age and sex; Model 2: + social class; Model 3: + physical activity, Mediterranean diet adherence, and smoking; Model 4: fully adjusted model.

**Figure 2.**
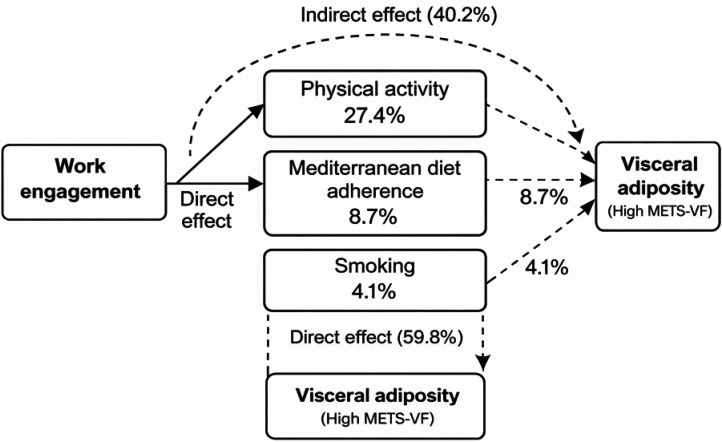
Mediation framework and the relative contribution of each lifestyle factor to the total effect.

The causal mediation analysis revealed that lifestyle behaviors accounted for a substantial proportion of the association between work engagement and visceral adiposity as measured by METS-VF. Overall, approximately 40% of the total effect of low versus very high work engagement on high METS-VF was mediated through the combined influence of physical activity, Mediterranean diet adherence, and smoking. Among these mediators, physical activity contributed the largest share of the indirect effect, followed by dietary adherence and smoking. Despite this attenuation, a significant direct effect of work engagement on visceral adiposity remained, indicating that additional non-behavioral mechanisms may also be involved.

The mediation framework and the relative contribution of each lifestyle factor to the total effect are illustrated in Figure 2, which depicts both the direct and indirect pathways linking work engagement to visceral adiposity.

### 3.3. Robustness and sensitivity analyses

**Figure 3.**
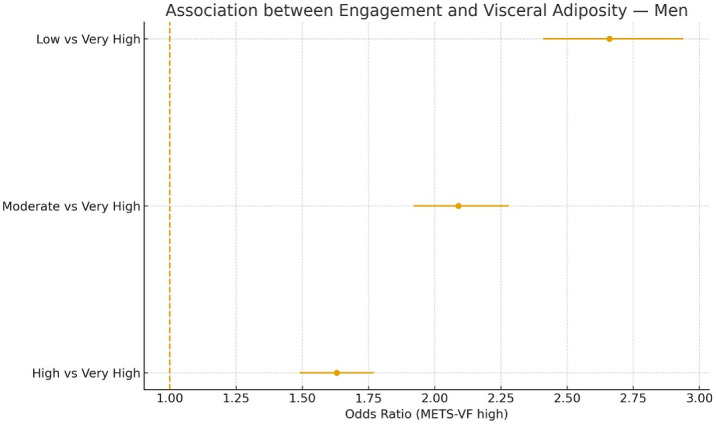
Association between engagement and visceral adiposity for men.

**Figure 4.**
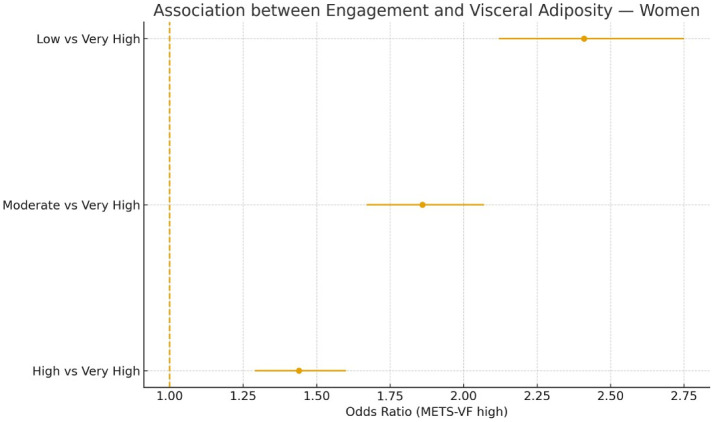
Association between engagement and visceral adiposity for women.

Sensitivity analyses confirmed the robustness of the findings across alternative outcome definitions (Table 4) and when adiposity indices were treated as continuous z-scores (Table 5). Subgroup analyses (Table 6) revealed slightly stronger associations in men, younger participants, and individuals with lower physical activity or poor adherence to the Mediterranean diet (Figures 3 and 4).

**Table 4.** Fully adjusted odds ratios by alternative outcome definitions.

Outcome	High OR (95% CI)	Moderate OR (95% CI)	Low OR (95% CI)	p-value
METS-VF high	1.56 (1.45–1.68)	1.99 (1.85–2.14)	2.59 (2.34–2.85)	<0.001
BMI obesity	1.44 (1.37–1.52)	1.92 (1.80–2.05)	2.30 (2.12–2.49)	<0.001
WtHR high	1.38 (1.30–1.47)	1.89 (1.77–2.01)	2.35 (2.14–2.57)	<0.001
CUN-BAE obesity	1.46 (1.39–1.54)	1.94 (1.83–2.06)	2.33 (2.16–2.52)	<0.001

Note: BMI, Body Mass Index. WtHR, waist-to-height ratio. CUN-BAE Clínica Universitária de Navarra Body Adiposity Estimator. METS-VF, Metabolic Score for Visceral Fat.

**Table 5.** Continuous specifications (z-score): linear/quantile regression (Model 4).

Outcome (z-score)	High β (95% CI)	Moderate β (95% CI)	Low β (95% CI)	p-value
METS-VF (z)	+0.18 (0.16–0.21)	+0.30 (0.27–0.33)	+0.42 (0.39–0.45)	<0.001
BMI (z)	+0.12 (0.10–0.15)	+0.23 (0.20–0.26)	+0.34 (0.31–0.37)	<0.001
WtHR (z)	+0.16 (0.14–0.19)	+0.28 (0.25–0.31)	+0.41 (0.38–0.44)	<0.001
CUN-BAE (z)	+0.14 (0.12–0.17)	+0.25 (0.22–0.28)	+0.36 (0.33–0.39)	<0.001

Note: BMI, Body Mass Index. WtHR, waist-to-height ratio. CUN-BAE, Clínica Universitária de Navarra Body Adiposity Estimator. METS-VF, Metabolic Score for Visceral Fat.

**Table 6.** Subgroup analyses for METS-VF (Model 4) and heterogeneity tests.

Stratum	High OR (95% CI)	Moderate OR (95% CI)	Low OR (95% CI)	p-interaction (vs overall)
Men	1.63 (1.49–1.77)	2.09 (1.92–2.28)	2.66 (2.41–2.94)	0.020
Women	1.44 (1.29–1.60)	1.86 (1.67–2.07)	2.41 (2.12–2.75)	0.020
<40 years	1.70 (1.53–1.89)	2.25 (2.03–2.49)	2.95 (2.62–3.32)	0.041
40–49 years	1.58 (1.43–1.75)	2.02 (1.83–2.23)	2.58 (2.31–2.89)	0.041
≥50 years	1.45 (1.31–1.60)	1.88 (1.70–2.07)	2.31 (2.04–2.61)	0.041
AF: Yes	1.46 (1.33–1.61)	1.89 (1.72–2.08)	2.41 (2.15–2.70)	0.032
AF: No	1.66 (1.51–1.84)	2.09 (1.90–2.31)	2.77 (2.48–3.09)	0.032
MedDiet: Yes	1.43 (1.30–1.58)	1.86 (1.68–2.05)	2.33 (2.07–2.61)	0.028
MedDiet: No	1.67 (1.51–1.84)	2.10 (1.91–2.32)	2.81 (2.51–3.14)	0.028

Note: BMI, Body Mass Index. WtHR, waist-to-height ratio. CUN-BAE, Clínica Universitária de Navarra Body Adiposity Estimator. METS-VF, Metabolic Score for Visceral Fat.

**Figure 5.**
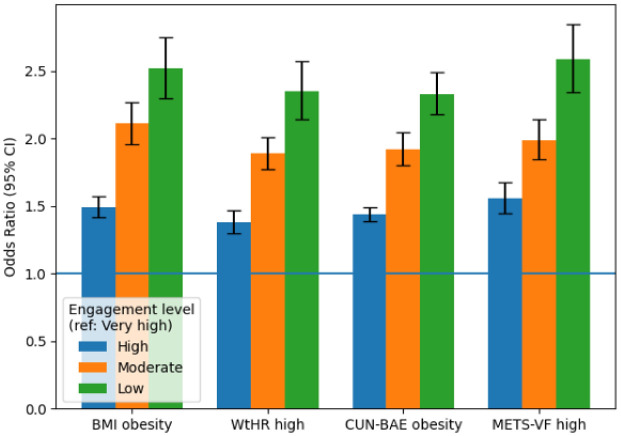
Dose–response of engagement across adiposity outcomes

Across all models and sensitivity checks, the associations remained significant after controlling for confounders, with consistent dose–response trends and p-trend values of <0.001 (Figure 5). Mediation analyses indicated that approximately 40% of the engagement–adiposity relationship was explained by differences in physical activity, diet, and smoking behaviors.

A detailed mediation analysis was performed using a parallel mediation model including physical activity, Mediterranean diet adherence, and smoking as mediators. Table S1 presents the complete model estimates, including the standardized regression coefficients for each path (a, b, c, and c'), indirect effects with bootstrapped 95% confidence intervals, and the proportion mediated by each behavioral factor.

### 3.4. Additional diagnostics and causal sensitivity analyses

Model calibration was adequate (slope ≈ 0.98; intercept ≈ −0.02; Brier score ≈ 0.083), and no influential observations were detected. Cluster-robust standard errors and mixed models with random intercepts by company yielded nearly identical ORs (ICC ≈ 0.012). Multiple imputation by chained equations (≥20 imputations) closely matched complete-case analyses. Control of false discovery rate (Benjamini–Hochberg) retained all key associations.

Figure S1 displays the overall forest plot for METS-VF (Model 4), Figure S2 shows the sex-stratified results, and Figure S3 depicts the calibration curve confirming model fit and discrimination.

Causal sensitivity analyses indicated high robustness: E-values for the engagement–METS-VF association were 2.49 (high), 3.39 (moderate), and 4.62 (low), suggesting that only very strong unmeasured confounders could nullify the observed relationships. Excluding current smokers did not materially change the results (low vs very high engagement = OR 2.47 [2.22–2.76]).

The hypothesized conceptual path model linking work engagement to visceral adiposity (METS-VF) through physical activity, Mediterranean diet adherence, and smoking is presented in Supplementary Figure S4.

In summary, lower levels of work engagement were consistently associated with higher odds of adiposity across all indices examined, with the strongest associations observed for visceral fat. These relationships followed a clear dose–response pattern and remained robust across multivariable adjustment, sensitivity analyses, and subgroup stratifications. Mediation analyses further demonstrated that lifestyle behaviors—particularly physical activity—partially explained the observed associations, underscoring their relevance as behavioral pathways linking work engagement to metabolic health.

## 4. Discussion

This study addresses an important gap in the literature by clarifying the association between work engagement and multiple indicators of adiposity, with particular emphasis on visceral fat, in a large occupational cohort. Our findings demonstrate a consistent dose–response relationship whereby lower levels of work engagement are associated with higher odds of adiposity across all indices examined, with the strongest and most robust associations observed for visceral adiposity as measured by METS-VF. Importantly, causal mediation analyses revealed that this relationship is partly explained by lifestyle behaviors, accounting for approximately 40% of the total effect, with physical activity emerging as the predominant mediator. These results position work engagement as a relevant psychosocial determinant of metabolic health and highlight modifiable behavioral pathways through which engagement may influence visceral fat accumulation.

With respect to our first hypothesis, which posited that lower work engagement would be associated with higher adiposity across multiple indices—particularly visceral adiposity—our results provide strong and consistent support for this assumption.

Although prior research directly examining the association between work engagement and visceral adiposity remains limited, existing studies typically integrate analyses of psychosocial work factors—such as health-related commitment, engagement, or stress—with outcomes related to obesity, BMI, or health behaviors [40]–[42]. Our findings provide novel and robust evidence that lower work engagement is strongly associated with higher levels of visceral fat in a large Spanish working population. Notably, this relationship was consistently stronger for METS-VF than for general anthropometric obesity indicators, suggesting that work engagement may be more closely linked to metabolically active fat depots than to overall body mass.

One plausible explanation is that higher engagement fosters sustained physical activity, healthier dietary patterns, and reduced smoking, as supported by our mediation results, which identified lifestyle behaviors—particularly physical activity—as key pathways. Beyond behavioral mechanisms, engagement may also influence visceral fat accumulation through psychosocial and biological processes, such as reduced chronic stress exposure, improved stress coping, and modulation of neuroendocrine responses, including hypothalamic–pituitary–adrenal axis activity and cortisol regulation [43]. Given the established link between visceral adiposity and cardiometabolic risk, these findings extend the occupational health literature by positioning work engagement as a relevant psychosocial factor with potential implications for metabolic regulation and disease prevention.

These results extend the psychosocial-metabolic literature by linking a positive work-related psychological state (engagement) with fat distribution phenotypes—especially visceral adiposity—which has greater pathophysiological relevance than general obesity [44]–[46].

While few prior studies have examined engagement per se in relation to obesity, some parallel evidence supports our findings. Sedentary behavior at work has been negatively associated with engagement levels and positively with adiposity measures [47]. This suggests that engaged workers may adopt more active postures or routines, reducing sedentary time and mitigating fat accumulation.

The link between psychological stress or low psychosocial resources and visceral fat has been documented in stress-biology research. For instance, chronic stress burden predicts increased visceral adipose tissue and inflammation [48]. Moreover, a recent meta-analysis on visceral adiposity and cardiovascular outcomes underscores the importance of visceral fat as a mediator of risk [49]. Our finding that engagement is more strongly associated with METS-VF than with BMI or WtHR aligns with such evidence that visceral adiposity may capture subtler metabolic burden not evident in crude anthropometry. In the neuroendocrine domain, stress-induced cortisol responses vary by BMI and can relate to adiposity [50]. Engagement may buffer stress pathways, reducing cortisol-driven lipogenesis, particularly in visceral fat depots. The intersection of engagement, stress regulation, and fat distribution remains largely unexplored, and our results contribute to bridging this gap.

Finally, research on digital health engagement and weight management emphasizes that sustained engagement is necessary for behavioral change [51]. While distinct from occupational engagement, these findings highlight behavioral pathways through which engagement could influence metabolic outcomes. Taken together, these findings confirm our first hypothesis and suggest that work engagement is more closely linked to metabolically relevant fat distribution than to general obesity measures.

These findings carry several implications. First, the workplace is a leverage point for preventive health interventions. Enhancing engagement, through job autonomy, recognition, social support, and purpose, could yield metabolic benefits beyond traditional health promotion [52]. Second, given the stronger link between engagement and visceral fat, occupational health programs might monitor indices beyond BMI, focusing on markers like METS-VF to evaluate intervention efficacy [53]. Third, engagement-enhancing strategies may offer dual benefits: improving psychological well-being and productivity while simultaneously reducing metabolic risk. Engagement could thus serve as a psychosocial biomarker for both performance and health [54],[55]. If the engagement–adiposity association proves causal, increasing engagement levels could contribute to modest but population-relevant reductions in visceral obesity burden, with downstream benefits in cardiovascular and metabolic disease prevention [56]. Building on this association, our second hypothesis proposed that lifestyle behaviors would partially mediate the relationship between work engagement and visceral adiposity. The mediation analyses directly support this hypothesis, demonstrating that physical activity, Mediterranean diet adherence, and smoking collectively explained a substantial proportion of the observed association.

This study opens several promising lines of inquiry. Longitudinal and interventional designs are needed to establish temporality and test whether improving engagement leads to reductions in visceral fat [57]. Mechanistic mediators beyond behavior, such as sleep quality, circadian disruption, inflammatory biomarkers, or neuroendocrine stress axes, should be explored to explain the residual direct effect observed in our mediation models [58]. Contextual moderators (industry, job type, hybrid vs. in-person work) may influence both engagement and metabolic outcomes. Furthermore, digital monitoring tools, such as wearables and ecological momentary assessment, could refine measurement of engagement and lifestyle factors in real time [59].

These directions can advance the field from cross-sectional associations toward actionable, mechanistically grounded interventions.

It is also important to consider the potential role of individual personality traits. Certain characteristics, such as high self-demand, conscientiousness, or perfectionistic tendencies, may extend work engagement to other domains of daily life and could therefore influence both psychosocial and behavioral pathways. Moreover, the association between engagement and physical activity may be bidirectional: while higher engagement could facilitate healthier behaviors, individuals who are more physically active may also experience enhanced well-being, energy, and engagement at work.

## 5. Study limitations

Several limitations of this study should be acknowledged when interpreting the findings. First, the cross-sectional design precludes causal inference and does not allow determination of the temporal direction of the observed associations between work engagement and adiposity. Although the analytical framework was guided by strong theoretical and empirical evidence, reverse causation cannot be ruled out, whereby individuals with poorer metabolic health or higher visceral adiposity may experience lower levels of work engagement.

Second, despite extensive adjustment for sociodemographic factors and key lifestyle behaviors, residual confounding from unmeasured variables remains possible. Factors such as sleep quality, work-related stressors outside the engagement construct, mental health conditions, personality traits, or non-occupational psychosocial stress were not available and may have influenced both engagement levels and adiposity outcomes.

Third, lifestyle behaviors, including physical activity, dietary habits, and smoking, were assessed using self-reported questionnaires. Although validated instruments were employed and administered under standardized conditions, reporting bias and social desirability bias cannot be entirely excluded. Such misclassification would likely be non-differential, potentially attenuating the observed associations and mediation effects.

Fourth, visceral adiposity was estimated using the METS-VF index, a validated and widely used surrogate marker that integrates anthropometric and metabolic parameters. While METS-VF shows strong correlations with imaging-based measures and cardiometabolic outcomes, it remains an indirect estimate and does not replace direct assessment of visceral fat by imaging techniques such as CT or MRI.

Finally, the study population consisted of Spanish workers attending mandatory occupational health examinations. Although the large sample size, nationwide coverage, and diversity of occupational sectors enhance external validity, the findings may not be fully generalizable to unemployed individuals, informal workers, or populations in different sociocultural or labor contexts.

Despite these limitations, the study is strengthened by its large sample size, use of validated instruments, comprehensive adjustment strategy, robust sensitivity analyses, and consistent dose–response patterns across multiple adiposity indices, supporting the reliability and relevance of the observed associations.

## 6. Conclusions

In this large Spanish occupational cohort, lower work engagement was consistently associated with higher adiposity across multiple indices, with the strongest associations observed for visceral fat as assessed by METS-VF. These relationships followed a clear dose–response pattern and remained robust after extensive adjustment and sensitivity analyses. Mediation analyses indicated that lifestyle behaviors—particularly physical activity, followed by diet and smoking—partially explained this association, accounting for approximately 40% of the total effect. Together, these findings highlight work engagement as a relevant psychosocial factor linked to metabolic health and suggest that workplace strategies aimed at enhancing engagement may contribute to the prevention of visceral obesity through favorable behavioral pathways.

## Use of AI tools declaration

The authors declare they have not used Artificial Intelligence (AI) tools in the creation of this article.


