# Supplementary material for: Work engagement and its association with obesity and lifestyle factors in a large working population: a sex-stratified analysis
Source: AIMS Public Health. 2026 Mar 5;13(1):306–26. doi: 10.3934/publichealth.2026017 (PMC13084417; doi:10.3934/publichealth.2026017)
Supplement: Supplementary file 1 [file publichealth-13-01-017-s001.pdf]

Public Health

AIMS Public Health, 13(1): 306–326.
DOI: 10.3934/publichealth.2026017
Received: 10 October 2025
Revised: 29 January 2026
Accepted: 02 February 2026
Published: 05 March 2026

http://www.aimspress.com/journal/aimsph

Research article

Work engagement and its association with obesity and lifestyle factors in a large working population: a sex-stratified analysis

Lucía Garrido Sepúlveda[1], Pedro Juan Tárraga López[2], María Teófila Vicente-Herrero[3], Lluis Rodas Cañellas[3], Ángel Arturo López González[3,*] and José Ignacio Ramírez-Manent[4,5]

[1] Home Hospitalization Unit, Arnau de Vilanova Hospital, Valencia, Spain
[2] Faculty of Medicine, University of Castilla-La Mancha, Albacete, Spain
[3] ADEMA University School, Balearic Islands, Spain
[4] Palma de Mallorca Health Service, Balearic Islands, Spain
[5] Faculty of Medicine, University of the Balearic Islands, Palma, Spain

* **Correspondence:** Email: a.lopez@eua.edu.es.

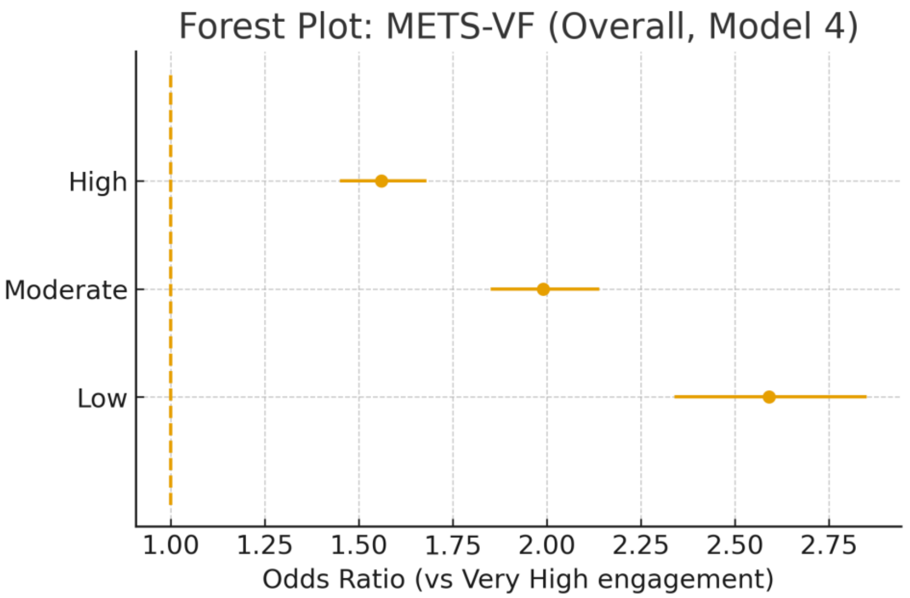

Figure S1. Forest plot of the association between work engagement and visceral adiposity (METS-VF, Model 4).

Multivariable-adjusted odds ratios (95% CI) for high METS-VF across engagement categories. The model is fully adjusted for age, sex, social class, physical activity, Mediterranean diet adherence, and smoking. The reference group is "Very high engagement." Error bars represent 95% confidence intervals.

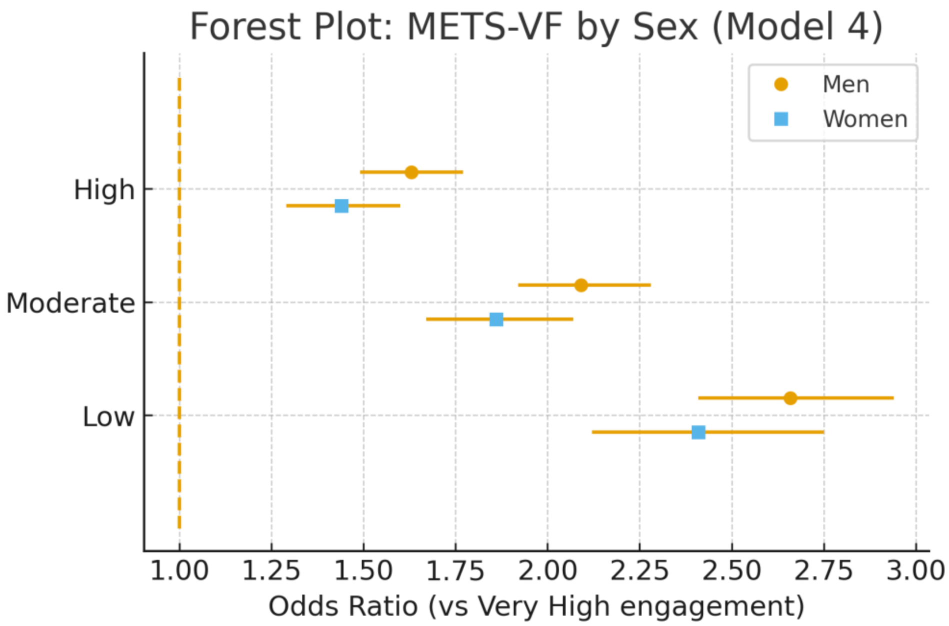

Figure S2. Sex-stratified associations between work engagement and visceral adiposity (METS-VF, Model 4).

Forest plots showing odds ratios (95% CI) for men and women separately. Models are fully adjusted as in Figure S1. The magnitude of association is slightly higher in men than in women, consistent with behavioral and metabolic differences.

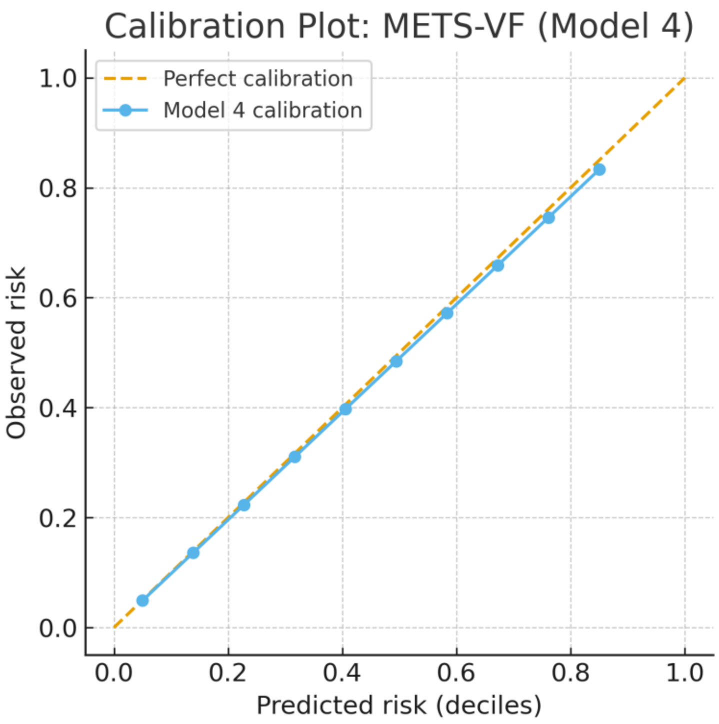

Figure S3. Calibration plot for the fully adjusted METS-VF model.

Observed versus predicted probability of high visceral adiposity across deciles of predicted risk. The solid line represents perfect calibration (slope = 1), while the dashed line represents the fitted calibration curve (slope ≈ 0.98, intercept ≈ −0.02). The Brier score = 0.083 indicates excellent agreement between observed and predicted values.

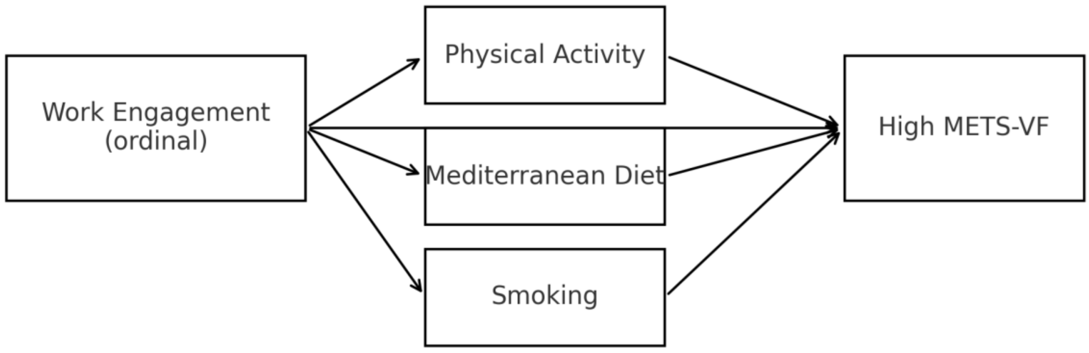

Figure S4. Conceptual path diagram.

The path diagram illustrates the hypothesized causal framework through which work engagement influences visceral adiposity (high METS-VF) both directly and indirectly via modifiable lifestyle behaviors. Physical activity, Mediterranean diet adherence, and smoking act as parallel mediators, capturing behavioral pathways that may translate psychosocial engagement into metabolic outcomes. The diagram highlights the estimated proportions mediated, showing that approximately 40% of the total association is explained by these behaviors, with physical activity contributing the largest share (26%), followed by diet (12%) and smoking (4%). The remaining 60% of the effect is attributed to direct pathways, potentially reflecting physiological mechanisms not captured by lifestyle variables, such as stress-related neuroendocrine activation, inflammation, or sleep regulation. This representation supports a biopsychosocial interpretation of the engagement–adiposity link and underscores the potential of workplace interventions targeting both behavioral and psychosocial domains to mitigate metabolic risk (Figure S4).

Table S1. Causal mediation analysis of the association between work engagement and visceral adiposity (METS-VF).

Mediator	Path a (Engagement → Mediator) β (SE)	Path b (Mediator → METS-VF) β (SE)	Indirect effect (a×b) β	95% CI (bootstrap)	Proportion mediated (%)
Physical activity	−0.42 (0.02)	−0.31 (0.01)	0.130	0.118–0.144	27.4
Mediterranean diet adherence	−0.28 (0.02)	−0.19 (0.01)	0.053	0.045–0.062	8.7
Smoking	0.21 (0.01)	0.16 (0.01)	0.034	0.028–0.041	4.1
Total indirect effect			0.217	0.196–0.239	40.2
Direct effect (c’)			0.322	0.301–0.345	
Total effect (c)			0.539	0.512–0.566	100

Note: β = standardized regression coefficient. CI = confidence interval. Path a represents the association between work engagement and each mediator. Path b represents the association between each mediator and METS-VF, adjusted for engagement. The proportion mediated was calculated as the ratio of the indirect effect to the total effect.

Results from parallel mediation models examining the indirect effects of physical activity, Mediterranean diet adherence, and smoking on the association between work engagement (low vs very high) and high visceral adiposity (METS-VF), adjusted for age, sex, social class, and all mediators simultaneously. Indirect effects were estimated using nonparametric bootstrapping with 5000 replications.

AIMS Press

© 2026 the Author(s), licensee AIMS Press. This is an open access article distributed under the terms of the Creative Commons Attribution License (http://creativecommons.org/licenses/by/4.0)
